# Interference in Macrophage Balance (M1/M2): The Mechanism of Action Responsible for the Anti-Inflammatory Effect of a Fluorophenyl-Substituted Imidazole

**DOI:** 10.1155/2024/9528976

**Published:** 2024-02-17

**Authors:** Julia Salvan da Rosa, Eduarda Talita Bramorski Mohr, Tainá Larissa Lubschinski, Guilherme Nicácio Vieira, Thais Andreia Rossa, Marcus Mandolesi Sá, Eduardo Monguilhott Dalmarco

**Affiliations:** ^1^Department of Clinical Analysis, Center for Health Sciences, Campus Universitário—Trindade, Universidade Federal de Santa Catarina, Florianópolis 88040-970, SC, Brazil; ^2^Department of Chemistry, Center for Physical and Mathematical Sciences, Campus Universitário—Trindade, Universidade Federal de Santa Catarina, Florianópolis 88040-970, SC, Brazil

## Abstract

Traditionally, the treatment of inflammatory conditions has focused on the inhibition of inflammatory mediator production; however, many conditions are refractory to this classical approach. Recently, an alternative has been presented by researchers to solve this problem: The immunomodulation of cells closely related to inflammation. Hence, macrophages, a critical key in both innate and acquired immunity, have been presented as an alternative target for the development of new medicines. In this work, we tested the fluorophenyl-imidazole for its anti-inflammatory activity and possible immunomodulatory effect on RAW 264.7 macrophages. We also evaluated the anti-inflammatory effect of the compound, and the macrophage repolarization to M2 was confirmed by the ability of the compound to reduce the M1 markers TNF-*α*, IL-6, MCP-1, IL-12p70, IFN-*γ*, and TLR4, the high levels of p65 phosphorylated, iNOS and COX-2 mRNA expression, and the fact that the compound was not able to induce the production of M1 markers when used in macrophages without lipopolysaccharide (LPS) stimulation. Moreover, fluorophenyl-imidazole had the ability to increase the M2 markers IL-4, IL-13, CD206, apoptosis and phagocytosis levels, arginase-1, and FIZZ-1 mRNA expression before LPS stimulation. Similarly, it was also able to induce the production of these same M2 markers in macrophages without being induced with LPS. These results reinforce the affirmation that the fluorophenyl-imidazole has an important anti-inflammatory effect and demonstrates that this effect is due to immunomodulatory activity, having the ability to trigger a repolarization of macrophages from M1 to M2a. These facts suggest that this molecule could be used as an alternative scaffold for the development of a new medicine to treat inflammatory conditions, where the anti-inflammatory and proregenerative properties of M2a macrophages are desired.

## 1. Introduction

Acute inflammation is a biological adaptive process that occurs naturally following an injury triggered by harmful external stimuli. Following trauma or infection, inflammation drives the restoration of homeostasis by protecting the host from exogenous pathogens and repairing damaged tissue [1, 2]. However, if this response is dysregulated, inflammation becomes uncontrolled, leading to the development and progression of various inflammatory and autoimmune diseases [3]. A dysregulated immune response can evolve to a catastrophic scenario, characterized by local or systemic tissue damage and excessive production of proinflammatory biomarkers [4, 5].

Macrophages play a pivotal role in the innate immune system, due to their plasticity and heterogeneity. Classically activated M1 macrophages express high levels of proinflammatory cytokines and large amounts of nitric oxide, while alternative activated M2 macrophages act during the inflammatory resolution phase, promoting tissue repair and the healing process. M2 macrophages are currently subdivided into M2a, M2b, and M2c subsets, with each presenting distinct characteristics and functions. Recently, some authors have called attention to new treatment strategies that seek to modulate the inflammatory resolution process by acting on macrophage polarization, offering an alternative to conventional anti-inflammatory approaches [6–8]. This new research has also attracted the attention of many pharmaceutical manufacturers, who are currently investing in developing new molecules capable of inducing macrophage polarization from profile M1 to M2, as an alternative approach to treat refractory inflammatory diseases and also some types of cancer [9, 10].

Among the new small molecules with anti-inflammatory effect, imidazole-containing compounds have shown the capacity to inhibit some pathways and biomarkers related to this process. Therefore, various imidazole-derivatives have been tested for their medical and clinical usefulness to treat various diseases and have demonstrated numerous biological activities, such as antifungal, antimicrobial, antiparasitic, anti-inflammatory, antihistamine, antibacterial, and anticancer effects [11, 12]. Current studies have reported promising anti-inflammatory activity of imidazole derivatives, based on their mechanism of action and important structure–activity relationship [13]. In regard to their anti-inflammatory activity, imidazole compounds have shown the capacity to decrease ciclooxigenase-2 (COX-2), phospholipase *A*2, lipoxygenase, TNF-*α*, IL-6, and nuclear factor-kappa B (NF-*κ*B) transcription [14, 15].

Our research group has previously demonstrated that fluorophenyl-imidazole presents prominent anti-inflammatory activity in murine *in vitro* and *in vivo* models, with no signs of acute oral toxicity in mice [16, 17]. Thus, the present study aims to determine whether the mechanism of the anti-inflammatory effect of fluorophenyl-imidazole (1-allyl-2-(4-fluorophenyl)-5-phenyl-1H-imidazole-4-methyl acetate) is linked to its ability to induce the change in macrophage profile, from the proinflammatory (M1) to anti-inflammatory or alternative (M2) phenotype.

## 2. Materials and Methods

### 2.1. The Compound 2-Fluorophenyl-Imidazole (Flu)

**Figure 1:**
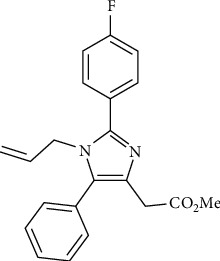
Chemical structure of the tetra substituted imidazole, methyl 1-allyl-2-(4-fluorophenyl)-5-phenyl-1H-imidazole-4-acetate.

The 2-fluorophenyl-imidazole used in this study was synthesized and provided by Dr. Thais Rossa, supervised by Professor Dr. Marcus Mandolesi Sá of the Department of Chemistry at the Universidade Federal de Santa Catarina (UFSC). The substituted fluorophenyl-imidazole (Figure 1) was synthesized by a reaction involving an azirine, a primary amine and an aldehyde [18]. The imidazole tested has a purity greater than 99% and was dissolved in 1% dimethyl sulfoxide (DMSO), following the predefined concentrations. After that, fluorophenyl-imidazole was aliquoted and stored at −80°C until the experiments, when it was properly dissolved in a cell culture medium.

### 2.2. Cell Culture

RAW 264.7 murine macrophage cells were purchased from the Rio de Janeiro Cell Bank (Rio de Janeiro, Rio de Janeiro, Brazil). The RAW 264.7 macrophages used for the tests were maintained in a controlled environment in a stove at 5% CO_2_ and at a temperature of 37°C. The culture medium used during the cultivation and experiments consisted of Dulbecco's Modified Eagle Medium (DMEM), supplemented with 10% fetal bovine serum and 1% antibiotics (streptomycin/penicillin). The experiments were performed between the third and eighth passages (80% cell confluence). The quantification of viable cells was performed by the Trypan blue technique, where all nonstained (viable) cells were counted in the four quadrants of a Neubauer chamber, using a common optical microscope (400x magnification).

### 2.3. Inflammatory Model

The *in vitro* inflammation model used lipopolysaccharide (LPS, 1 *µ*g/mL) to achieve the required inflammatory response. Depending on the experiment, RAW 264.7 macrophages were seeded in 6-, 12-, 24-, and 96-well plates and incubated for 4 or 24 hr. Next, the cell culture medium was replaced, and the cells were pretreated following predefined concentrations and group divisions, for 30 min, with subsequent induction with LPS. After the hours necessary for incubation, depending on the experiment, the cells or supernatants were collected, and these were used to measure the pro- and anti-inflammatory parameters. The cell division groups were: blank control (a), represented by noninflamed cells, cells pretreated with vehicle only (1% DMSO); (b) characterized by inflamed LPS cells pretreated with vehicle; (c) with inflamed cells pretreated with dexamethasone (7 *µ*M)—a reference anti-inflammatory plus LPS; and treatment group (d) represented by pretreated cells with predefined concentrations of fluorophenyl-imidazole (*n* = 3/group) with or without LPS. The only group not induced with LPS was the blank control (A), which received sterile PBS.

### 2.4. Cell Viability (Cytotoxicity Test)

The effect of the 2-fluorophenyl-imidazole on cell viability was evaluated using the Resazurin assay [19]. RAW 264.7 cells were seeded in 96-well plates with a cell density of 5 × 10^4^ cells/well. Subsequently, the cells were treated with the 2-fluorophenyl-imidazole at predefined concentrations (1, 3, 10, 30, 100, 300, and 1,000 *µ*M) and the blank control, and incubated for a further 24 hr. The test used a Rezarsurin solution (1.5 mg/mL) diluted in culture medium in contact with pretreated cells for 2 hr, with subsequent reading in fluorescence, using a wavelength of 530/590 nm in a Geminis™ XPS microplate spectrofluorometer (Molecular Devices, CA, USA).

Based on these results, it was possible to determine the CC_10_ value for the 2-fluorophenyl-imidazole, i.e., the concentration capable of killing 10% of the cell population, and consequently maintaining a viability of 90% [20, 21]. This parameter was calculated through nonlinear regression analysis of the concentration logarithm as a function of the normalized response (percentage of cell viability) using GraphPad Prism® version 8.0 (San Diego, CA, USA).

### 2.5. Concentration of Nitric Oxide Metabolites (NO*x*)

NO production was measured indirectly through the Griess reaction [22]. In this test, the IC_50_ of each compound tested was determined, i.e., the concentration of the compound capable of inhibiting 50% of the nitric oxide metabolites when compared with the inflamed group (negative control) [23, 24].

### 2.6. Cytokines by Flow Cytometry (L-12p70, TNF-*α*, IFN-*γ*, MCP-1, IL-6, and IL-10)

Cells were plated and treated with controls and with the 2-fluorophenyl-imidazole at NO*x* IC_50_ (1 *μ*M). After 30 min, they were stimulated with LPS (1 *μ*g/mL), except for the blank control. The supernatant was used to determine the concentrations of the cytokines IL-12p70, TNF-*α*, IFN-*γ*, MCP-1, IL-6, and IL-10 (pg/mL) using the cytometric bead array (CBA)–cell inflammation kit (BD) (Biosciences, San Diego, CA, USA), by flow cytometry, using the FacsVerse® Flow Cytometer (BD Biosciences, San Diego, CA, USA).

### 2.7. Dosage of Cytokines by ELISA (IL-4 and IL-13)

Cells were plated and treated with the controls and the best dose of the 2-fluoropheny-imidazole (1 *μ*M). After 30 min, they were stimulated with LPS (1 *μ*g/mL), except for the blank control. The levels of cytokines IL-4 and IL-13 were measured using the supernatant with commercial kits (PREPOTECH, Rocky Hill, New Jersey, USA) by the immunoenzymatic method (ELISA), following the manufacturer's instructions. Optical densities were determined at a wavelength of 450 nm in a microplate reader (ELISA MB-580, HEALES, Gouwei Road, SZN, China), and the results were expressed in pg/mL.

### 2.8. Evaluation of P–P_65_ NF-*κ*B Phosphorylation

Macrophages were plated in a 12-well plate. After confluence, cells were treated with controls and fluorophenyl-imidazole (1 *μ*M). After 30 min, they were stimulated with LPS (1 *μ*g/mL), except for the blank control. Cells were collected in an Eppendorf tube and washed with sterile PBS. Afterward, they were transferred to ELISA microplates containing monoclonal antibodies specific against the phosphorylated p_65_ protein (PathScan®Phospho-NF-*κ*B p65 (Ser536) ELISA Kit (Cell Signalling Technology, Inc., Danvers, Massachusetts, USA)). The experimental protocol was carried out following the manufacturer's guidelines, using the total cell extract (cytoplasmic and nuclear fractions). The results were expressed as the result of the blank control group, which represented the basal expression of phosphorylated p65 protein.

### 2.9. Evaluation of Macrophage Apoptosis

Cells were plated and treated with controls and fluorophenyl-imidazole (1 *μ*M) and after a 30-min wait, they were stimulated with LPS (1 *μ*g/mL). Taxel (Paclitaxel®-Merck, Darmstadt, Germany) at 30 *μ*M was used alone in one group of experiments, as positive control of apoptosis. Afterward, the cells were collected and used to determine cellular apoptosis levels using the FITC Annexin V Apoptosis Detection Kit (BD Biosciences). The experiment followed the protocol and instructions provided by the kit manufacturer. Samples were read in a FacsVerse® flow cytometer (BD Biosciences, São José, CA, USA).

### 2.10. Macrophage Phagocytosis

Cells were plated and treated with fluorophenyl-imidazole (1 *µ*M) and controls, and after 30 min, were stimulated with LPS (1 *μ*g/mL). After incubation, 100 *μ*L/well of neutral red dye solution (0.075%) was added, and the plate was incubated for another hour (37°C). The macrophages were then washed with PBS solution. One hundred microliters per well of cell lysis buffer (1% glacial acetic acid: ethanol; 1 : 1) was added, and the plate was incubated for an additional 1 hr at room temperature. The absorbance reading was performed in an ELISA reader MB-580 (HEALES, Gouwei Road, SZN, CN) at 540 nm. The results were expressed as the phagocytic index, which was measured using the following equation: phagocytic index = *A*1/*A*0, where *A*1 is the absorbance of the sample, LPS or dexamethasone, and *A*0 is the absorbance of the blank control.

### 2.11. Evaluation of the Expression of Mannose (CD206^hi+^) and TLR4 (CD284-MD2) Cell Receptors

The macrophages were pretreated with the 1 *μ*M concentration of fluorophenyl-imidazole and controls. After, the macrophages were stimulated with LPS (1 *µ*g∕mL). After collecting and washing the cells with PBS and discarding the supernatant, 1% albumin was added, followed by incubation for 1 hr and 30 min. The CD206 (FITC) or CD284-MD2 (PE) antibody was added, followed by incubation in the dark for 15 min. The CD206^hi+^ was measured using mouse anti-CD206 mouse kit (BD Biosciences, San Jose, California, USA) and CD284-MD2 was measured using BD Pharmingen rat antimouse complex (BD Biosciences, San Jose, California, USA). Their expression was evaluated by flow cytometry (BD Bioscience FACVerse® Flow Cytometer, San Jose, California, USA). The results were quantified as the percentage of receptors with expression using the FACS Suite® software program (BD Biosciences, San Jose, California, USA).

### 2.12. RT-qPCR Quantification of mRNA Expression of iNOS, COX-2, FIZZ-1, and Arginase-1

After pretreatment, cells were induced with LPS and incubated for 4 hr in 6-well plates (5 × 10^5^ cells/well). RAW 264.7 macrophages were scraped and centrifuged (200 g, 5 min, 4°C) for RNA purification by column method extraction, using a commercial kit (Zymo Research Corp., Irvine, California, USA). The RNA concentration was determined by measuring the absorbance at 260 nm using a NanoVue® Plus UV–Vis Spectrophotometer (Chicago, Illinois, USA). The extracted RNA was treated with DNase, and then added to a solution containing Oligo(dT), RevertAid® H minus M-MULV Reverse Transcriptase, Ribolock RNase Inhibitor® and dNTP mix (Thermo Fisher Scientific, Waltham, MA, USA), according to the manufacturer's protocol. All incubations were performed with thermocycler equipment (MJ Research-Bio-Rad, Hercules, CA, USA). The RT-qPCR reaction was performed with 50 ng of cDNA, with specific primers to amplify the genes of interest (NOS_2_, COX-2, ARG-1, and FIZZ-1) added to a Ludwig Biotec® SYBR Green qPCR master mix qPCR/ROX master mix (2x) (Ludwig Biotec, Alvorada, Rio Grande do Sul, Brazil). Samples were transferred to 96-well qPCR plates (StepOnePlus®, Applied Biosystems, Carlsbad, CA, USA). A melting curve analysis was performed to verify the specificity of each pair of primers. Samples were compared using the relative CT method in comparison with the (S) group results (2^−*ΔΔ*CT^). The primer sequences are as follows: GAPDH (sense: 5′-GTG.TCC.GTC.GTG.GAT.CTG.AC-3′, antisense: 5′-GGA. GAC.AAC.CTG.GTC.CTC.AG-3′), iNOS (sense: 5′-CGA. AGT.TTC.TGG.CAG.CAG.C-3′, antisense: 5′-AGC. ACT.CTC.TTG.CGG.ACC.AT-3′), COX-2 (sense: 5′-CAA. AGG.CCT.CCA.TTG.ACC.GA-3′, antisense: 5′-TGG. ACG.AGG.TTT.TTC.CAC.CAG-3′), Arginase-1 (sense: 5′-GTT.CCC.AGA.TGT.ACC.AGG.ATT.C-3′, antisense: 5′-CGA.TGT.CTT.TGG.CAG.ATA.TGC-3′), and Fizz-1 (sense: 5′-CCA. ATC.CAG.CTA.ACT.ATC.CCT.AC-3′, antisense: 5′-ACC. CAG.CAG.CAG.TCA.TCC.CA-3′).

### 2.13. Reagents and Drugs

The following drugs and reagents used were obtained from BD Biosciences (San Diego, California, USA): IL-1*β*, IL-4, and IL-13 ELISA kit, Annexin V-FITC, PE Rat IgC2a, K Isotype Control, PE Rat Anti-Mouse CD284/MD-2 (TLR-4 marker), FITC Rat Anti-Mouse CD-206 (Mannose marker), 7-AAD (propidium iodide), cytometric bead array—CBA Mouse inflammation kit, Folin & Ciocalteu's phenol reagent; Gibco (Grand Island, New York, USA): DMEM, DMEM without phenol, fetal bovine serum (FBS), Penicillin–Streptomycin (10,000 U/mL), Versene® (2 g EDTA-Na4), Tripan blue dye; LaborClin (Pinhais, Paraná, Brazil): phosphate-buffered saline; Invitrogen (Massachusetts, USA): Neutral red; MTT (3-(4,5- dimethylthiazol-2-yl)-2,5-Diphenyltetrazolium Bromide); Cell Signaling Technology (Massachusetts, USA): NF-*κ*B p65 (Ser536) ELISA kit; LabSynth (Diadema, São Paulo, Brazil): ethanol, trisodium citrate, copper sulfate, double tartrate Na/K, sodium hydroxide; Newprov (Pinhais, Paraná, Brazil): Panotic® dye; Peprotech (Rocky Hill, New Jersey, USA): Mouse TNF-*α* and IL-6 ELISA kits; Rio de Janeiro Cell Bank (Rio de Janeiro, Rio de Janeiro, Brazil): RAW 264.7 macrophages; Sigma–Aldrich Co. (St. Louis, Missouri, USA): lipopolysaccharide O111:B4 (*Escherichia coli*), dexamethasone (minimum 98% HPLC), resazurin, *α*-naphthylethylenediamide.2HCl, phenol, sodium azide, sodium dodecylsulfate, sodium hypochlorite, sulfanilamide; Thermo Fisher Scientific (Massachusetts, USA): Oligo(dT), RevertAid® H minus M-MULV Reverse Transcriptase, Riboblock RNase Inhibitor®, dNTP mix; Ludwig Biotec (Alvorada, Rio Grande do Sul, Brazil): SYBR Green qPCR/ROX master mix (2x), primers (iNOS, COX2), nuclease-free water; Syntec (Hortolândia, São Paulo, Brazil): xylazine hydrochloride (2%), ketamine hydrochloride (10%); Vetec (Rio de Janeiro, Rio de Janeiro, Brazil): dimethyl sulfoxide, polysorbate-20 (5%), sodium hydrogen phosphate, and zinc sulfate. Other reagents used but not listed above were obtained from alternative commercial sources.

### 2.14. Statistical Analysis

The results were analyzed using GraphPad Prism® version 8.0 (San Diego, California, USA). The experimental results were expressed as mean ± standard error of the mean (SEM). All the data were considered parametric homoscedastic by one-way ANOVA followed by the Tukey post hoc test. Significance was set at *P*  < 0.05.

## 3. Results

### 3.1. Cytotoxicity (Cell Viability)

**Figure 2:**
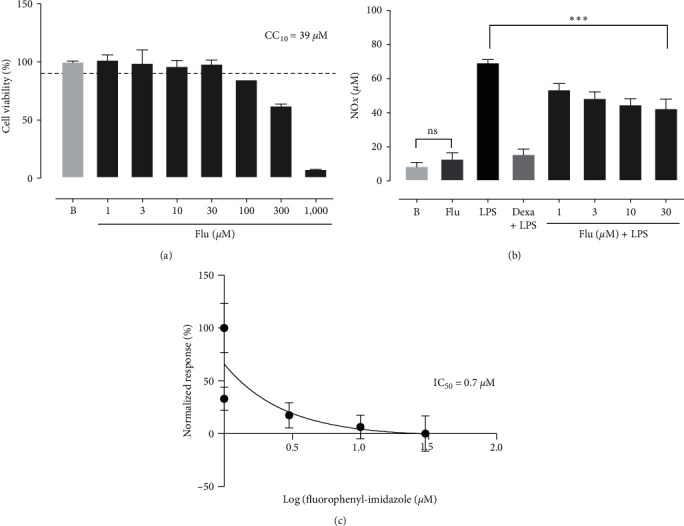
Cell viability (cytotoxicity) and NO*x* (*µ*M) inhibition of fluorophenyl-imidazole on macrophage RAW 264.7. The cytotoxicity assay (a) was performed using the resazurin colorimetric assay, where RAW 264.7 macrophages were treated with 1–1,000 *μ*M of fluorophenyl-imidazole (Flu). After 24 hr, the cell viability (CC_10_) was determined. For the NO*x* assay (b), cells were treated with vehicle or pretreated with dexamethasone (Dexa, 7 *µ*M) or flu at the indicated concentrations and then treated with LPS (1 *μ*g/mL), or they were treated with LPS alone (1 *μ*g/mL). The sigmoid curve (c) represents de IC_50_ of imidazole fluorophenyl and shows the normalized response of the methyl 1-allyl-2-(4-fluorophenyl)-5-phenyl-1H-imidazole-4-acetate on NO*x* metabolite levels in RAW 264.7 macrophages. All the experiments were performed in triplicate. The results were expressed as the mean ± SEM and the IC_50_ for fluorophenyl-imidazole were calculated by nonlinear regression analysis using the logarithm of concentration vs. normalized response. ns, not significant;  ^*∗∗∗*^*P*  < 0.001.

Fluorophenyl-imidazole exhibited significant cytotoxicity only at high concentrations (up to 100 *µ*M) (Figure 2(a)). Moreover, the calculated CC_10_ for this 2-fluorophenyl-imidazole was 39 *µ*M, ensuring a safe concentration of 90% cell viability during the experiments (Figure 2(a)). To maintain cell integrity, we decided to use only concentrations below 30 *µ*M in the subsequent experiments.

### 3.2. Effect of Fluorophenyl-Imidazole on NO*x* Levels

NO*x* inhibition was measured to evaluate the anti-inflammatory activity of fluorophenyl-imidazole using the concentrations under the CC_10_ value (30, 10, 3, and 1 *µ*M). Fluorophenyl-imidazole was able to inhibit NO*x* production at all the concentrations tested (30, 10, 3, and 1 *µ*M) (inhibition (%): 45.0 ± 4.2; 42.3 ± 2.8; 37.5 ± 3.0; 30.7 ± 2.7) (*P*  < 0.001) (Figure 2(b)). As expected, dexamethasone also inhibited the levels of this inflammatory mediator at the tested concentration (7 *µ*M) (inhibition (%): 79.9 ± 1.5) (*P*  < 0.001) (Figure 2(b)). The NO*x* IC_50_ was defined as 0.7 *µ*M, and all the subsequent experiments were conducted using a concentration of 1 *µ*M (Figure 2(c)).

### 3.3. Quantification of Pro- and Anti-Inflammatory Cytokines

When the macrophages were pretreated with fluorophenyl-imidazole used at 1 *µ*M, before LPS, the levels of all the proinflammatory cytokines tested were significantly reduced in relation to the LPS group: (percentage of inhibition for TNF-*α*: 30.7 ± 2.7 (*P*  < 0.01); IL-6: 35.6 ± 5.3 (*P*  < 0.001); MCP-1: 26.3 ± 4.0 (*P*  < 0.001); IL-12p70: 36.0 ± 7.4; (*P*  < 0.05) IFN-*γ*: 8.2 ± 2.0 (*P*  < 0.05) (Table 1). However, when the macrophages were treated with fluorophenyl-imidazole only, the levels of these cytokines did not change in comparison with untreated cells (blank) (*P*  > 0.05) (Table 1). Furthermore, 2-fluorophenyl-imidazole, at the same concentration as before LPS stimulation, increased the production of the anti-inflammatory cytokines IL-10, IL-4, and IL-13 in relation to the LPS group (percentage increase for IL-10: 68.4 ± 3.7; IL-4: 14.7 ± 4.2; IL-13: 35.7 ± 7.8) (*P*  < 0.05) (Table 1). Surprisingly, the treatment of macrophages with fluorophenyl-imidazole, without LPS stimulation produced a significant increase in the levels of all studied anti-inflammatory cytokines when compared with untreated cells (blank) (percentage increase for IL-10: 55.6 ± 1.2 (*P*  < 0.05); IL-4: 35.9 ± 1.7 (*P*  < 0.001); IL-13: 78.4 ± 2.4 (*P*  < 0.01) (Table 1).

### 3.4. Effect of Fluorophenyl-Imidazole on TLR4 Receptor and Mannose Receptor (CD206)

**Figure 3:**
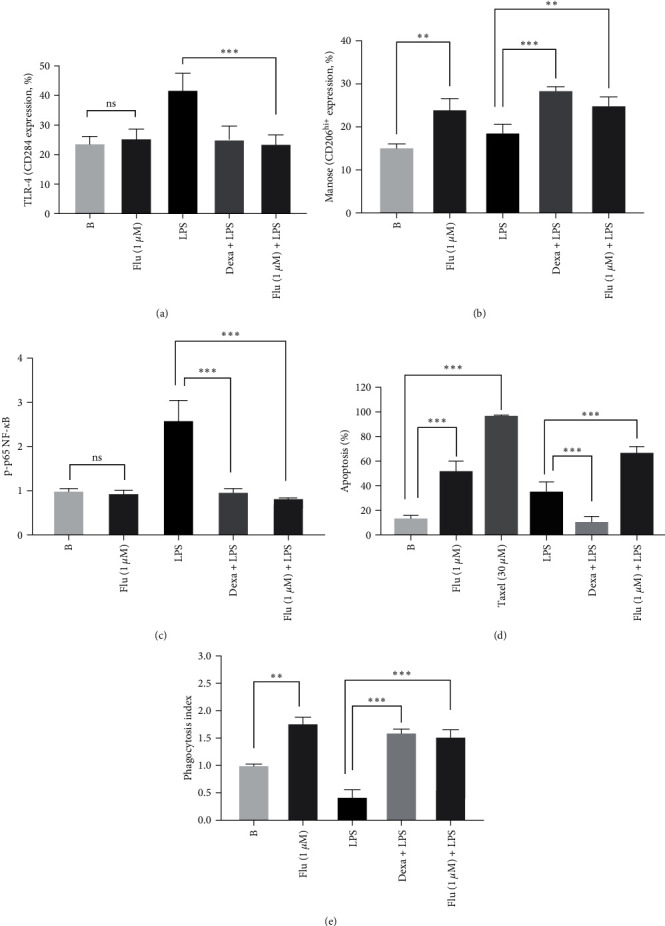
Effect of fluorophenyl-imidazole on TLR-4 receptor (CD284-MD2) (a); mannose receptor (CD206^hi+^) (b); phosphorylated protein p65 (c); apoptosis (d); and phagocytic activity (e) in RAW 264.7 macrophages stimulated with LPS (1 *μ*g/mL). The experimental groups used were B: cells pretreated with vehicle and incubated with PBS (blank group); LPS: cells pretreated with vehicle and stimulated with LPS (1 *μ*g/mL); dexa + LPS: cells pretreated with dexamethasone (7 *μ*M) and stimulated with LPS (1 *μ*g/mL); flu (1 *μ*M): cells pretreated with the best concentration (based on IC_50_ values) (1 *µ*M) of fluorophenyl-imidazole alone; and (flu 1*μ*M) + LPS: fluorophenyl-imidazole (1 *µ*M), before LPS (1 *μ*g/mL). Taxel (30 *μ*M) was used alone in apoptosis experiments, as positive control of apoptosis. The results for TLR-4 (CD284-MD2) and (CD206^hi+^) were quantified as receptor expression percentage in relation to cells stained with control isotype antibody while, phosphorylated protein p65 expression was compared to the blank control (cells with no treatment) that represents the basal expression phosphorylated p65 protein. The apoptosis values (%) were quantified as the percentage of apoptotic cells compared with the total cell number and the phagocytic index was measured using the following equation: phagocytic index (PI) = *A*1/*A*0; where *A*1 is the absorbance of sample, LPS and dexamethasone, and *A*0 is the absorbance of black control. The results were expressed as the mean ± SEM; *n* = 3; ns, not significant;  ^*∗∗*^*P*  < 0.01; and  ^*∗∗∗*^*P*  < 0.001.

Fluorophenyl-imidazole at 1 *µ*M before LPS stimulation was capable of decreasing the expression of TLR-4 receptor (CD284-MD2) when compared with the LPS control group (inhibition (%): 44.0 ± 4.7) (*P*  < 0.001) (Figure 3(a)). However, when the macrophages were treated with fluorophenyl-imidazole alone, without LPS stimulation, the expression of the TLR-4 receptor (CD284-MD2) was not changed when compared with the untreated cells (blank) (*P*  > 0.05) (Figure 3(a)). Dexamethasone before LPS stimulation decreased this parameter (inhibition (%): 39.6 ± 5.0) (*P*  < 0.001) (Figure 3(a)).

Furthermore, the 2-fluorophenyl-imidazole used before LPS, increased the expression of mannose receptor (CD 206^hi+^) on the surface of the macrophages RAW 264.7 in comparison to the LPS group (increase (%): 34.3 ± 5.0) (*P*  < 0.01) (Figure 3(b)). The fluorophenyl-imidazole used alone in the treatment of the macrophages was able to increase the expression of this cell receptor (CD 206^hi+^) when compared with the blank control group (*P*  < 0.01) (Figure 3(b)). The treatment of cells with dexamethasone, before LPS stimulation, also increased CD206 expression in relation to the LPS group (53.4 ± 2.5) (*P*  < 0.001) (Figure 3(b)).

### 3.5. Quantification of Phosphorylated Protein p65 (NF-*κ*B)

We performed the quantification of phosphorylated protein p65 to evaluate the capacity of fluorophenyl-imidazole to inhibit the NF-*κ*B activation. The 2-fluorophenyl-imidazole at 1 *µ*M, before LPS, decreased the phosphorylation of the p65 subunit in relation to the LPS group (inhibition (%): 68.4 ± 0.4) (*P*  < 0.001) (Figure 3(c)). When macrophages were treated with fluorophenyl-imidazole without LPS stimulation, the levels of phosphorylated protein p65 were similar to those of the blank group (*P*  > 0.05) (Figure 3(c)). The treatment with dexamethasone before LPS, also significantly inhibited this inflammatory parameter compared with the LPS group (inhibition (%): 63.0 ± 2.2) (*P*  < 0.001) (Figure 3(c)).

### 3.6. Effect of Fluorophenyl-Imidazole on Macrophage Apoptosis and Phagocytic Activity

Fluorophenyl-imidazole (1 *µ*M), before LPS stimulation, produced a significant increase in the levels of macrophage apoptosis when compared to the LPS group (increase (%): 90.1 ± 8.3) (*P*  < 0.001) (Figure 3(d)). In macrophages treated only with fluorophenyl-imidazole, without LPS stimulation, the levels of apoptosis were significantly increased in comparison with the blank group (*P*  < 0.001) (Figure 3(d)). Conversely, in the group pretreated with dexamethasone before LPS stimulation, the levels of apoptosis were significantly reduced in comparison with the LPS group (*P*  < 0.001) (Figure 3(d)).

Fluorophenyl-imidazole, used before LPS stimulation, increased the phagocytic activity when compared with the LPS group (increase (%): 257.2 ± 18.0) (*P*  < 0.001) (Figure 3(e)). Fluorophenyl-imidazole alone was able to induce the phagocytic activity of the macrophages when compared with the blank group (increase (%): 73.5 ± 8.4) (*P*  < 0.01) (Figure 3(e)). The reference drug dexamethasone, before LPS stimulation, also increased the phagocytic activity compared with the LPS group (increase (%): 287.1 ± 7.2) (*P*  < 0.001) (Figure 3(e)).

### 3.7. RT-qPCR Quantification of mRNA Expression of iNOS, COX-2, Arginase-1, and FIZZ

**Figure 4:**
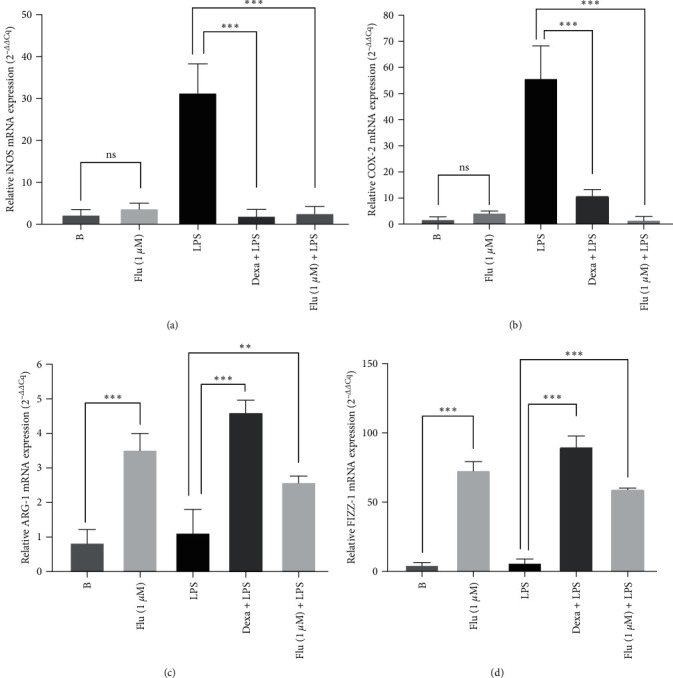
Effect of fluorophenyl-imidazole on iNOS (a); COX-2 (b); ARG-1 (c); and FIZZ-1 (d) mRNA expression in RAW 264.7 macrophages stimulated with LPS (1 *μ*g/mL). The experimental groups used was B: cells pretreated with vehicle and incubated with PBS (blank group); LPS: cells pretreated with vehicle and stimulated with LPS (1 *μ*g/mL); dexa: cells pretreated with dexamethasone (7 *μ*M) and stimulated with LPS (1 *μ*g/mL); flu: fluorophenyl-imidazole: cells pretreated with the best concentration (based on IC_50_ values) (1 *µ*M) of fluorophenyl-imidazole alone, and flu (1 *μ*M) + LPS: cells pretreated with the best concentration (based on IC_50_ values) (1 *µ*M) of fluorophenyl-imidazole and stimulated with LPS (1 *μ*g/mL). iNOS, COX-2, ARG-1, and FIZZ-1 mRNA were calculated in duplicate by relative CT method in comparison to the (S) group results (2^−*ΔΔ*CT^). The results were expressed as the mean ± SEM; *n* = 3;  ^*∗∗*^*P*  < 0.01; and  ^*∗∗∗*^*P*  < 0.001.

In these experiments, fluorophenyl-imidazole (1 *µ*M), used before LPS stimulation, produced massive inhibition of mRNA expression of iNOS (inhibition (%): 92.3 ± 2.4) (*P*  < 0.001) (Figure 4(a)), and mRNA expression of COX-2 in relation to the LPS group (inhibition (%): 97.3 ± 1.7) (*P*  < 0.001) (Figure 4(b)). The anti-inflammatory control, dexamethasone, used before LPS stimulation inhibited these parameters (percentage of inhibition iNOS: 94.3 ± 2.8; COX-2 : 80.3 ± 2.6) (*P*  < 0.001) (Figures 4(a) and 4(b)). The fluorophenyl-imidazole used alone, without LPS stimulation, was not able to increase the mRNA expression of both iNOS and COX-2 when compared with the blank group (*P*  > 0.05) (Figures 4(a) and 4(b)).

Meanwhile, fluorophenyl-imidazole before LPS stimulation was capable of increasing the mRNA expression of Arginase-1 (increase (%):132.7 ± 10.5) (*P*  < 0.01) (Figure 4(c)) and the mRNA expression of FIZZ-1 (increase (%): 1,006.0 ± 13.1) (*P*  < 0.001) (Figure 4(d)) in relation to the LPS group. Dexamethasone, also used before LPS stimulation, increased Arginase-1 (increase (%): 317.7 ± 19.1) (*P*  < 0.001) (Figure 4(c)) and FIZZ-1 (increase (%): 1,577.0 ± 90.0) (*P*  < 0.001) (Figure 4(d)) in relation to the LPS group. Otherwise, the fluorophenyl-imidazole, used without LPS stimulation, produced a massive induction of mRNA expression of both Arginase-1 and FIZZ-1 when compared with the blank group (percentage of inhibition Arginase-1 : 431.3 ± 27.1 and FIZZ-1 : 1892.3 ± 256.9) (*P*  < 0.001) (Figures 4(c) and 4(d)).

## 4. Discussion

The drugs currently used to treat inflammatory conditions are steroids (NSAIDs) and corticosteroids, but these can have important local and systemic side effects [25, 26]. To try and solve this problem, the pharmaceutical industry has been investigating new compounds, including proton pump inhibitors and antihelminthics that have been synthesized by optimization of imidazole derivatives [27, 28]. Previously, our research group demonstrated the fluorophenyl-imidazole exhibits anti-inflammatory effect, inhibiting nitric oxide metabolites and proinflammatory cytokine secretion in LPS-induced J774 macrophages. We subsequently confirmed this anti-inflammatory activity using two different lung injury models *in vivo*. These results proved that fluorophenyl-imidazole has the ability to inhibit leukocyte migration, myeloperoxidase, nitric oxide metabolite, and cytokine secretion. We also found that fluorophenyl-imidazole inhibited p38 MAPK and NF-*κ*B phosphorylation, with no signs of acute oral toxicity [16, 17]. However, until now, the underlying mechanism that orchestrates this effect is still unknown. Therefore, in this work, we attempt to link the anti-inflammatory effect of fluorophenyl-imidazole to the possible immunomodulation/polarization of macrophages, since our previous experiments showed that 2-fluorophenyl-imidazole was able to inhibit the proinflammatory cytokines, and at the same time, enhanced the levels of cytokines with anti-inflammatory profile [16, 17].

In the present work, we first tested the capacity of fluorophenyl-imidazole to inhibit nitric oxide and proinflammatory cytokine secretion after LPS stimulation at noncytotoxic concentration, in this cell lineage. The cytotoxicity assay was performed and showed low cytotoxicity, which is consistent with other research using LPS-induced RAW 264.7 macrophages [29, 30]. Fluorophenyl-imidazole was capable of inhibiting nitric oxide metabolites and proinflammatory cytokines. Other studies tested imidazole-containing compounds as a potential anti-inflammatory agent *in vitro* and found that it inhibits oxide nitric and several proinflammatory cytokines [31–33]. Imidazole compounds present structural affinity to bind and inhibit oxide nitric production due to structural similarity to the heme-ligand type that carries an imidazole or related heterocyclic donor group [34, 35].

Much of the current research to test the anti-inflammatory effect of some molecules has focused on the inhibition of COX-1 and COX-2 as modifying agents of the inflammatory process. Since 2014, several studies have reported new COX-2 inhibitors containing imidazole and imidazoline moieties [36, 37]. Supporting this affirmation, research has demonstrated the decrease of PE2 levels and COX-2 gene inhibition. These results corroborate with the COX-2 inhibition caused by fluorophenyl-imidazole treatment in our experiments.

The increase in the production of proinflammatory biomarkers is directly correlated with the LPS-activated TLR4–NF-*κ*B signaling pathway. We observed that LPS-induced RAW 264.7 macrophages treated with fluorophenyl-imidazole showed a significant decrease in p65 phosphorylation and TLR-4 (CD284-MD2) expression, resulting in NF-*κ*B inhibition. Our finding is supported by Baek et al. [38] and Li et al. [39], who designed a similar LPS-induced RAW 264.7 macrophage experiment to prove that the anti-inflammatory activity of imidazole-containing compounds is directly related to their ability to decrease TLR-4 expression and p65 phosphorylation.

In parallel with the fluorophenyl-imidazole anti-inflammatory activity, as already mentioned above, our research group evaluated the possible proresolution effect of fluorophenyl-imidazole through immunomodulation of LPS-induced macrophages. In these experiments, fluorophenyl-imidazole was able to increase anti-inflammatory cytokine production directly related to M2 macrophage polarization, upregulating IL-4, IL-10, and IL-13 release. Srivastava et al. [40] reported that TRIM (1-(2-trifluoromethylphenyl) imidazole) has a similar ability to regulate anti-inflammatory cytokines release, promoting macrophage polarization (M1 to M2 phenotypes) in LPS-induced RAW 264.7 macrophages. Furthermore, another study using primary cultures of macrophages treated with an imidazole derivative demonstrated a shift from the M1 proinflammatory phenotype to the M2 subpopulation, with decreased levels of proinflammatory mediators and increased levels of anti-inflammatory cytokines [41]. Moreover, the M2 macrophage phenotype increased the expression of nuclear factors such as FIZZ-1, Arginase-1, Ym1, as well as surface receptors such as mannose receptor (CD206), which are related to the increase in phagocytic ability. These biomarker expressions are involved in parasite infestation, tissue remodeling, and tumor progression (immunoregulatory functions) [42, 43]. The fluorophenyl-imidazole studied was able to increase the CD206 expression, suggesting an enhancement of the M2 macrophage population. Moreover, this molecule also increased the mRNA expression of Arginase-1 and FIZZ-1. Other imidazole-containing compounds showed the ability to upregulate Arginase-1 expression in LPS-induced bone marrow-derived macrophages (BMM) [41]. Interestingly, our experiments demonstrated that fluorophenyl-imidazole was able to increase the phagocytic capacity in LPS-induced RAW 264.7 macrophages, maintaining their apoptotic levels high, to more characteristics of M2 macrophages. Findings indicate that the inflammatory reaction is heading toward the resolution phase. In fact, these findings are very exciting since the fluorophenyl-imidazole used alone, also demonstrated the same profile. At the same time, this compound was not able to induce production or stimulate M1 markers. Results that allow us to reinforce the affirmation that this compound has an immunomodulatory profile on Raw 264.7 macrophages. Therefore, these results qualify the fluorophenyl-imidazole as possible scaffold to develop new drugs with anti-inflammatory effect with immunomodulatory mechanism of action.

Due to the plasticity of macrophages, all populations (M1 and M2 subsets M2a, M2b, and M2c) can be found the focus on inflammation. However, after the treatment with fluorophenyl-imidazole, we can hypothesize that the major macrophage population in our *in vitro* experimental model will be represented by M2a macrophage subtypes, characterized by the increase in M2a phenotype markers (IL-4, IL-13, IL-10, CD206, FIZZ-1, and Arg-1) [44–46]. Also, FIZZ-1 expression is considered a specific biomarker of the M2a subpopulation in murine and human macrophages [47–49].

## 5. Conclusion

Fluorophenyl-imidazole showed distinctive anti-inflammatory *in vitro* activity, decreasing all the proinflammatory cytokines tested, NO*x* and TRL-4 (D284-MD2 receptors) and causing expressive inhibition of p65 NF-*κ*B translocation. Furthermore, fluorophenyl-imidazole was capable of increasing IL-4, IL-13, IL-10, CD206, FIZZ-1, Arginase-1, and phagocytic activity, demonstrating unprecedented immunomodulatory activity with macrophage repolarization (from M1 to M2) and exhibiting an increase in M2a macrophage subpopulation, suggesting a protective activity by leading the inflammatory reaction toward the resolution phase.

## Figures and Tables

**Table 1:** Effect of fluorophenyl-imidazole on the production of pro- and anti-inflammatory cytokines.

Cytokines	B (only vehicle 1%)	LPS (1 *µ*g/mL)	Dexa (7 *µ*M) + LPS	Flu (1 *µ*M)	Flu (1 *µ*M) *+* LPS
Proinflammatory					
TNF-*α*	1,447.0 ± 156.2	187,429.0 ± 8,371.0	71,350.0 ± 8, 430.0^*∗∗∗*^	1,441.0 ± 100.2	129,845.0 ± 5, 183.0^*∗∗*^
IL-6	9.9 ± 0.2	45.2 ± 2.479	36.2 ± 0.6 ^*∗*^	10.3 ± 0.7	29.1 ± 2.4 ^*∗∗∗*^
MCP-1	224.8 ± 2.0	6,443.0 ± 144.2	5,648.0 ± 124.6 ^*∗*^	271.2 ± 3.7	4,748.0 ± 252.0 ^*∗∗∗*^
IL-12p70	8.0 ± 0.4	8.7 ± 0.4	5.4 ±0.8^*∗*^	8.3 ± 0.9	5.5 ± 0.6 ^*∗*^
IFN-*γ*	6.6 ± 0.2	7.8 ± 0.1	7.3 ± 0.1 ^*∗∗*^	7.1 ± 1.0	7.3 ± 0.1 ^*∗*^
Anti-inflammatory					
IL-10	11.5 ± 0.8	9.7 ± 2.0	18.5 ± 1.9 ^*∗∗*^	17.9 ± 1.2^#^	16.4 ± 0.4 ^*∗*^
IL-4	1,140.0 ± 38.5	1,211.0 ± 46.3	1,653 ± 56.8 ^*∗∗∗*^	1,550.0 ± 18.2^###^	1,390.0 ± 51.2 ^*∗*^
IL-13	431.8 ± 12.5	512.2 ± 32.8	800.4 ± 34.7 ^*∗∗*^	771.8 ± 9.5^##^	695.1 ± 39.9 ^*∗*^

The effect of fluorophenyl-imidazole (flu) on the pro- and anti-inflammatory cytokines measured by flow cytometry/CBA (TNF-*α*, IL-6, MCP-1, IL-12p70, IFN-*γ*, and IL-10) and ELISA (IL-4 and IL-13). Both results were expressed in pg/mL. The experimental groups used was B: cells pretreated with vehicle and incubated with PBS (blank group); LPS: cells pretreated with vehicle and stimulated with LPS (1 *μ*g/mL); dexa: cells pretreated with dexamethasone (7 *μ*M) and stimulated with LPS (1 *μ*g/mL); flu: fluorophenyl-imidazole: cells pretreated with the best concentration (based on IC_50_ values) (1 *µ*M) of fluorophenyl-imidazole alone, and flu (1 *μ*M) + LPS: cells pretreated with the best concentration (based on IC_50_ values) (1 *µ*M) of fluorophenyl-imidazole and stimulated with LPS (1 *μ*g/mL). Each measurement was performed in triplicate, and each group represents the mean of five samples. The results were expressed as the mean ± SEM of the absolute values (pg/mL).  ^*∗*^*P*  < 0.05,  ^*∗∗*^*P*  < 0.01, and  ^*∗∗∗*^*P*  < 0.001 (compared with the LPS group). ^#^*P*  < 0.05, ^##^*P*  < 0.01, ^###^*P*  < 0.001 (compared with the blank group).

## Data Availability

The data used to support the findings of this study are included in the article.
